# Differences in Telemedicine Use Between Rural and Urban Medicare Beneficiaries With Kidney Failure

**DOI:** 10.1016/j.xkme.2026.101329

**Published:** 2026-03-20

**Authors:** Joel T. Adler, Arnold E. Kuk, Layla Parast

**Affiliations:** 1 Division of Transplantation, Department of Surgery and Perioperative Care, Dell Medical School at the University of Texas at Austin, Austin, TX; 2 Biomedical Data Sciences Hub, Dell Medical School at the University of Texas at Austin, Austin, TX; 3 Department of Statistics and Data Science, University of Texas at Austin, Austin, TX

**Keywords:** End-stage kidney disease, digital access, health disparities, Medicare, mental health services, rural health, telehealth, telemedicine, virtual care

## Abstract

**Rationale & Objective:**

Telemedicine offers a potential solution to address disparities in health care access among rural and urban patients with kidney failure. This study examined telehealth utilization patterns and urban–rural differences before and during the coronavirus disease 2019 (COVID-19) pandemic.

**Study Design:**

Retrospective observational cohort study.

**Setting & Population:**

Medicare beneficiaries (with both parts A and B) with kidney failure, on kidney replacement therapy of any modality (excluding transplant) from the United States Renal Data System, totaling 787,959 patients (2017-2021).

**Exposure:**

Urban versus rural residence, before (January 2020) and during (January 2021) the COVID-19 pandemic.

**Outcomes:**

Telehealth utilization, defined as any claim for telemedicine services, including mental health, primary care, nephrology, and other services.

**Analytical Approach:**

Multivariable logistic regression assessed predictors of telehealth use. Geographic variation was analyzed using county-level data.

**Results:**

Rural patients were more likely to use telehealth prepandemic (OR, 4.61; 95% CI, 4.11-5.28; *P* < 0.01), but urban patients had higher utilization during the pandemic (OR, 139.55; 95% CI, 128.33-153.94; *P* < 0.01). The relative increase in telehealth use was greater among urban patients (urban–rural interaction: OR, 0.16; 95% CI, 0.14-0.18; *P* < 0.01) than before pandemic. African American (OR, 0.73; 95% CI, 0.72-0.75; *P* < 0.01) patients were less likely to use telehealth than White patients. Telehealth use was more common among patients receiving peritoneal dialysis (OR, 1.37; 95% CI, 1.34-1.41; *P* < 0.01) or in-center self-hemodialysis (OR, 1.59; 95% CI, 1.26-1.97; *P* < 0.01) compared with in-center hemodialysis. Minimal differences in service types were observed between rural and urban patients.

**Limitations:**

Exclusion of Medicare Advantage and private insurance data may limit generalizability. The study did not assess outcomes associated with telehealth use.

**Conclusions:**

Telehealth use considerably increased for patients with kidney failure during the pandemic, particularly in urban areas, but racial, ethnic, and social disparities persist. Efforts to promote equitable telehealth use and access are essential to advance health equity for patients with kidney failure.

## Plain-Language Summary

During the COVID-19 pandemic, many health care providers began using telehealth to care for patients remotely. For people receiving dialysis for kidney failure, telemedicine offered a safer and more convenient way to communicate with their care team. In this study, we looked at how telemedicine was used among adults with Medicare who were receiving dialysis, comparing patterns in rural and urban areas. We found that overall use was higher in rural areas, largely because of increased mental health services. These findings suggest that telemedicine plays an important role in making health care more accessible, but future policies must support technology access and digital literacy to ensure all patients can benefit.

Geographic disparities in access to care are well documented between urban and rural patients with kidney failure in the United States.^1^^,^^2^ Compared with their urban counterparts, the 240,000 rural dwellers with kidney failure are less likely to start dialysis under the care of a nephrologist,^3^^,^^4^ are less likely to finish kidney transplant evaluation,^5^ and are less likely to be waitlisted for kidney transplantation.^6^ Addressing these access challenges is crucial to improving equity in care delivery. Telehealth, with its ability to facilitate remote patient–provider interactions, is well positioned to overcome the geographic barriers faced by rural patients with kidney failure.

Telehealth is broadly defined as the “use of electronic information and telecommunication technologies to support long-distance clinical healthcare,” with telemedicine referring specifically to its clinical care subset.^7^^,^^8^ The adoption of telehealth^8^^,^^9^ expanded dramatically during the coronavirus disease 2019 (COVID-19) pandemic,^10^^,^^11^ facilitated by temporary regulatory waivers and reimbursement expansions.^12^ Even before the pandemic, telemedicine use among rural Medicare beneficiaries was growing steadily, particularly for primary care and telemental health services.^10^^,^^13^^,^^14^ This expansion was supported by key policy changes. The Bipartisan Budget Act of 2018 allowed patients on home hemodialysis or peritoneal dialysis to conduct 2 of 3 monthly visits via telehealth regardless of location, starting in 2019. During the COVID-19 emergency, additional federal waivers lifted geographic and site restrictions, broadened provider eligibility, and enhanced reimbursement.^15^ Many of these flexibilities were extended via waiver into 2023 and 2024, highlighting how policy directly shaped telemedicine access and uptake for patients with kidney failure.

In contrast, telehealth’s adoption in dialysis care has been less consistent. Qualitative studies of in-center hemodialysis patients found that while telemedicine offered greater convenience, patients also reported concerns about privacy and the limitations of remote examinations.^16^^,^^17^ Evidence from a qualitative meta-analysis further supports continued telemedicine for patients with chronic kidney disease and kidney transplant recipients to improve access to care.^18^ However, among patients with kidney failure, significant regional variation in nephrologists’ telehealth utilization has been observed during and after the COVID-19 pandemic.^19^

Telemedicine has played an increasing role in chronic disease management, particularly for conditions requiring continuous monitoring, such as diabetes, hypertension, and congestive heart failure. Patients with diabetes benefited significantly from telemedicine in reducing hospitalization rates and improving glycemic control, whereas patients with heart failure experienced reduced readmissions.^10^ However, its adoption among patients with kidney failure has been more complex, given the frequent need for in-person dialysis treatments. Understanding how patients with kidney failure engage with telemedicine—and whether patterns resemble those in other chronic conditions—may help clarify its potential benefits in this population.

There is less known about the geographic variation in telehealth utilization and how rural and urban patients with kidney failure, who are treated with dialysis and have not undergone kidney transplantation, differ in their use of telemedicine before and during the COVID-19 pandemic. Using Medicare claims data for patients treated with dialysis from 2017-2021, we aimed to (1) describe telehealth utilization from 2017-2021 among rural and urban patients with kidney failure; (2) identify patient characteristics associated with a higher likelihood of being a telehealth user both before (January 2020) and during (January 2021) the COVID-19 pandemic, with a specific focus on comparing urban and rural patients; and (3) assess geographic variation in telemedicine use both before (January 2020) and during (January 2021) the COVID-19 pandemic. These findings aim to inform strategies for improving care delivery to rural patients with kidney failure.

## Methods

### Data Sources and Institutional Review Board Approval

The data reported here have been supplied by the United States Renal Data System (USRDS). The interpretation and reporting of these data are the responsibility of the authors and in no way should be seen as an official policy or interpretation of the US government.^20^ Race and ethnicity were recorded by either the patient or their dialysis provider on the CMS-2728 form at dialysis initiation. We obtained the number of COVID-19 cases by county at specific time points from the *New York Times* database.^21^

The Institutional Review Board of the University of Texas at Austin approved the limited use data set (STUDY00002098); because this was a secondary analysis of data, no informed consent was required. This manuscript complies with Strengthening the Reporting of Observational Studies in Epidemiology guidelines for cohort studies.^22^

### Study Cohort

The study cohort included patients who had Medicare parts A and B, as indicated in the PAYHIST file, in any given month from 2017-2021. We excluded patients whose initial kidney failure service fell under one of the following categories: uncertain dialysis status, discontinued dialysis, kidney transplant, loss to follow up, recovered kidney function, or missing data. Additionally, patients with incomplete information required for the models in the analysis were excluded. This resulted in a primary study cohort of 787,959 patients. The secondary study cohort was a subset of the primary study cohort and consisted only of patients who had Medicare claims in January 2020 and/or January 2021 (440,250 patients).

### Variables

Our primary variable of interest was telemedicine utilization by patients with kidney failure. Telemedicine visits were defined as visits with place of service code of 02 (telemedicine) or with any one of the following modifiers: 95 (synchronous telemedicine service rendered via a real-time interactive audio and video communications system), GQ (telehealth service rendered via asynchronous telecommunications systems), or GT (telehealth service rendered via interactive audio and video telecommunications systems). A patient was considered to be a telehealth user in January 2020 if they had ≥1 telehealth claim in January 2020. Similarly, a patient was considered to be a telehealth user in January 2021 if they had ≥1 telehealth claim in January 2021.

Covariates included age (categorized as 0-19, 20-29, 30-39, 40-49, 50-59, 60-69, 70-79, and ≥80 years), sex (male or female), race (White, African American, Native American/American Indian, Native Hawaiian/Pacific Islander, or other), ethnicity (Hispanic or non-Hispanic), primary disease (hypertension, glomerulonephritis, cystic disease, urologic, other, unknown, or missing), last observed kidney replacement therapy modality (in-center self-hemodialysis, in-center hemodialysis, home hemodialysis, or peritoneal dialysis; this was updated with most recent CMS-2728 for each month), employment status (full-time, medical leave of absence, part-time, retired, student, unemployed, or other), institutionalized (yes or no), Centers for Disease Control and Prevention/Agency for Toxic Substances and Disease Registry (CDC/ATSDR) social vulnerability index (ranked in quartiles; higher quartiles are more vulnerable counties), and type of rural-urban commuting area (urban or rural).

We classified claims by using both the specialty of the provider and the diagnosis code as mental health, nephrology, primary care, and other. Physician claims were assigned by their primary specialty; claims from physician assistants and nurse practitioners were defined by diagnosis codes (Table S1) because they do not have a primary specialty assigned in Medicare claims. We defined the Medicare insured population as patients who had ≥1 Medicare claim identified in the USRDS files in the month of analysis. This means that our analysis is restricted to the population of patients with kidney failure who filed Medicare claims, whether or not Medicare was their primary insurer.

### Statistical Analysis

We calculated visit rates per 1,000 patients by dividing the total number of telehealth visits in each category (eg, primary care and mental health) by the number of patients eligible for that service in a given month and multiplying by 1,000. Rates for subgroups (eg, rural and urban patients; service utilization) were calculated separately using their respective denominators; these stratified rates reflect within-group utilization and are not additive to the overall rate.

We used a multivariable logistic regression model to identify patient characteristics associated with a higher likelihood of being a telehealth user both before (January 2020) and during (January 2021) the COVID-19 pandemic, with a specific focus on comparing urban versus rural patients. The outcome was whether a patient used telemedicine in the particular month, ie, the variable was equal to 1 if the patient had ≥1 telemedicine claim in the particular month and 0 otherwise. The predictors in the model were urban versus rural patient address, pre-COVID–19 (January 2020) versus during COVID-19 (January 2021), an interaction between these 2 predictors to examine whether urban–rural differences changed during COVID-19 compared with pre-COVID–19, as well other patient characteristics including age, sex, race, ethnicity, primary disease, first kidney replacement therapy modality, employment status, institutionalization status, and CDC/ATSDR social vulnerability index. The CDC/ATSDR social vulnerability index was used as a proxy for county-level social disadvantage, categorized into quartiles, in which lower quartiles represent more vulnerable counties.

When displaying this data on a map (January 2020 and January 2021), we used the residential county from the PATIENTS table, which is updated with the most recent CMS-2728 form available with each annual USRDS release. Counties with <11 residents with kidney failure were left blank. Because a moderate number of counties also had 0 telehealth claims for those months, we calculated the absolute change in telehealth users per 1,000 residents with kidney failure rather than the relative change.

To account for the potential influence of residing in a high-COVID–19-outbreak county on telemedicine use, we conducted a sensitivity analysis. An indicator variable was created to identify patients from counties with high-COVID–19 outbreak rates. In January 2020, 87% of patients resided in counties with no COVID-19 outbreaks. Therefore, the 90th percentile of COVID-19 outbreak rates (0.0004% in 2020, 28.81% in 2021) was used as the threshold to define high-outbreak counties, and this same threshold was applied to data from 2021.

Given that some patients were in the dataset for both January 2020 and January 2021 (ie, paired data) whereas others were not, we used bootstrapping to appropriately estimate standard errors. An alpha level of 0.05 was used as criterion for statistical significance. All statistical analyses and data linkages were performed using SAS 9.3. Figures were produced with R 4.0.4 (R Core Team), and maps were created in ArcGIS Pro 3.2.2 (ESRI).

## Results

### Descriptive Trends in Telehealth Utilization Over Study Period

**Figure 1:**
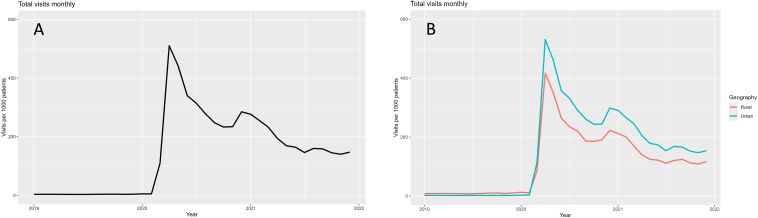
Telehealth utilization over time. Changes in telehealth utilization over time for all patients (A) and stratified for urban and rural patients (B) from 2019-2021. Utilization peaked for all patients shortly after the first incident of coronavirus disease 2019 (COVID-19) in 2021 and slowly decreased through the end of 2021.

Telehealth utilization was relatively low before the pandemic (Fig 1). In January of 2020, there were 4.9 visits per 1,000 patients with kidney failure in the entire sample (Fig 1A) and 12.8 visits per 1,000 rural patients with kidney failure compared with 3.1 visits for urban patients with kidney failure (Fig 1B). At peak usage in April 2020, there were 510.0 visits per 1,000 patients overall, with 417.2 for rural and 530.7 for urban patients. By the end of 2021, there were 147.4 visits per 1,000 patients overall, with 111.7 for rural and 154.1 for urban patients. These represent stratified rates per 1,000 patients within each geographic category and are not expected to sum to an overall rate, as each is calculated using a different subgroup denominator.

In January of 2020, per 1,000 patients with kidney failure, there were 0.4 visits for primary care, 1.9 visits for mental health, 1.2 visits for nephrology, and 1.3 visits for other services. At peak usage in April 2020, there were 116.4 visits for primary care, 39.7 visits for mental health, 158.2 visits for nephrology, and 195.8 visits for other services. At the end of 2021, there were 30.5 visits for primary care, 27.4 visits for mental health, 38.7 visits for nephrology, and 50.9 visits for other services.

**Figure 2:**
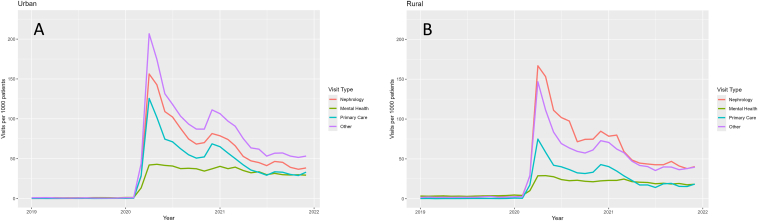
Telehealth utilization by service type among rural and urban patients. Changes in telehealth utilization by service type for urban (A) and rural (B) patients from 2019-2021. The majority of the services used were in “other” categories, followed by primary care, nephrology-related, and mental health claims. Classifications of “other” service types are available in Table S1.

We then analyzed these changes by service type, considering urban and rural differences. For urban patients in January 2020 (Fig 2A), there were 0.3 visits for primary care, 1.3 visits for mental health, 0.5 visits for nephrology, and 1.1 visits for other services. At peak usage in April 2020, there were 125.6 visits for primary care, 42.1 visits for mental health, 156.3 visits for nephrology, and 206.7 visits for other services. At the end of 2021, there were 33.1 visits for primary care, 29.3 visits for mental health, 38.4 visits for nephrology, and 53.3 visits for other services.

For rural patients in January 2020 (Fig 2B), there were 0.8 visits for primary care, 4.6 visits for mental health, 4.8 visits for nephrology, and 2.5 visits for other services. At peak usage in April 2020, there were 74.9 visits for primary care, 28.8 visits for mental health, 166.8 visits for nephrology, and 146.7 visits for other services. At the end of 2021, there were 18.4 visits for primary care, 18.3 visits for mental health, 40.3 visits for nephrology, and 39.6 visits for other services.

### Characteristics Associated With Telemedicine Use

The pre-COVID–19 (January 2020) cohort included 370,901 patients. Of these, 1,035 (0.28%) had an identified claim for telehealth use. The post-COVID-19 (January 2021) cohort included 316,397 patients; 57,205 (18.1%) had a claim for telehealth utilization. The breakdown of the percentage of telehealth utilization versus no utilization by each characteristic within each cohort is shown in Table 1 (with row percentages available in Table S2).

**Table 1:** Characteristics of Users and Nonusers of Telehealth, Before and During COVID-19

Covariate	January 2020				January 2021			
	User (N = 1,035)		Non-User (N = 369,866)		User (N = 57,205)		Non-User (N = 259,192)	
	n	%	n	%	n	%	n	%
County RUCA code								
Urban	503	48.6%	302,917	81.9%	48,628	85.0%	210,389	81.2%
Rural	532	51.4%	66,949	18.1%	8,577	15.0%	48,803	18.8%
Age (y)								
0-19	25	2.4%	6,269	1.7%	1,227	2.1%	4,480	1.7%
20-29	42	4.1%	16,955	4.6%	3,217	5.6%	12,087	4.7%
30-39	96	9.3%	33,599	9.1%	5,856	10.2%	23,314	9.0%
40-49	169	16.3%	57,596	15.6%	9,028	15.8%	38,839	15.0%
50-59	234	22.6%	84,993	23.0%	12,618	22.1%	56,911	22.0%
60-69	245	23.7%	90,821	24.6%	13,672	23.9%	64,152	24.8%
70-79	158	15.3%	57,014	15.4%	8,498	14.9%	42,383	16.4%
≥80	66	6.4%	22,609	6.1%	3,089	5.4%	17,026	6.6%
Sex								
Female	490	47.3%	158,611	42.9%	26,264	45.9%	108,809	42.0%
Male	545	52.7%	211,255	57.1%	30,941	54.1%	150,383	58.0%
Race								
African American	245	23.7%	123,079	33.3%	37,256	65.1%	84,138	32.5%
Native American/American Indian	60	5.8%	5,163	1.4%	831	1.5%	3,758	1.5%
Native Hawaiian, Pacific Islander	8	0.8%	4,194	1.1%	692	1.2%	2,973	1.2%
Other	27	2.6%	17,299	4.7%	3,311	5.8%	12,708	4.9%
White	695	67.2%	220,131	59.5%	37,256	65.1%	155,615	60.0%
Ethnicity								
Hispanic or Latino	95	9.2%	54,303	14.7%	9,494	16.6%	36,590	14.1%
Not Hispanic or Latino	940	90.8%	315,563	85.3%	47,711	83.4%	222,602	85.9%
Cause of kidney failure								
Cystic disease	35	3.4%	13,547	3.7%	2,435	4.3%	9,836	3.8%
Diabetes mellitus	501	48.4%	159,385	43.1%	24,494	42.8%	108,447	41.8%
Glomerulonephritis	109	10.5%	44,037	11.9%	7,980	14.0%	30,841	11.9%
Hypertension	233	22.5%	108,404	29.3%	14,550	25.4%	77,515	29.9%
Urologic	22	2.1%	6,705	1.8%	1,032	1.8%	4,935	1.9%
Other	111	10.7%	29,497	8.0%	5,264	9.2%	20,994	8.1%
Unknown	23	2.2%	8,242	2.2%	1,440	2.5%	6,574	2.5%
Missing cause	—a	—a	—a	—a	—a	—a	50	0.02%
First kidney replacement therapy modality								
Home hemodialysis	18	1.7%	10,312	2.8%	2,401	4.2%	6,897	2.7%
In-center self-hemodialysis	—a	—a	346	0.1%	100	0.2%	223,175	86.1%
In-center hemodialysis	939	90.7%	321,337	86.9%	46,074	80.5%	295	0.1%
Peritoneal dialysis	77	7.4%	37,871	10.2%	8,630	15.1%	28,825	11.1%
Employment status								
Full time	101	9.8%	48,804	13.2%	8,470	14.8%	35,472	13.7%
Medical leave of absence	35	3.4%	16,214	4.4%	2,668	4.7%	11,288	4.4%
Other	37	3.6%	13,616	3.7%	2,231	3.9%	8,920	3.4%
Part-time	35	3.4%	12,468	3.4%	2,070	3.6%	8,852	3.4%
Retired	549	53.0%	173,247	46.8%	25,994	45.4%	122,539	47.3%
Student	25	2.4%	4,172	1.1%	870	1.5%	2,952	1.1%
Unemployed	253	24.4%	101,345	27.4%	14,902	26.1%	69,169	26.7%
Institutionalized	90	8.7%	14,707	4.0%	2,094	3.7%	9,825	3.8%
County social vulnerability index								
Quartile 1 (<0.25) [high]	169	16.3%	34,462	9.3%	5,389	9.4%	26,008	10.0%
Quartile 2 (≥0.25, <0.50)	258	24.9%	74,307	20.1%	12,177	21.3%	53,258	20.6%
Quartile 3 (≥0.50, ≤0.75)	212	20.5%	100,365	27.1%	15,352	26.8%	70,460	27.2%
Quartile 4 (≥0.75) [low]	396	38.3%	160,732	43.5%	24,287	42.5%	109,466	42.2%

Abbreviations: COVID-19, coronavirus disease 2019; RUCA, rural-urban commuting area.

a Values suppressed if the number of individuals is <11 in a cell, or if it could be calculated from those values.

Adjusted logistic regression results examining characteristics associated with telemedicine use are shown in Table 2. Pre-COVID–19, being a patient in a rural county was associated with a higher likelihood of being a telehealth user (odds ratio [OR], 4.61; 95% confidence interval [CI], 4.11-5.28; *P* < 0.01) compared with patients residing in an urban county. The onset of COVID-19 significantly increased the likelihood of being a telehealth user, but this effect varied depending on the urban/rural status of the county of residence (OR, 0.16; 95% CI, 0.14-0.18; *P* < 0.01 for interaction). Among urban patients, the onset of COVID-19 significantly increased the likelihood of being a telehealth user (OR, 139.55; 95% CI, 128.33-153.94; *P* < 0.01), while the effect of COVID-19 on rural patients was still large but less pronounced (OR, 22.08; 95% CI, 20.21-24.12; *P* < 0.01). In January 2021, the odds of a rural patient being a telehealth user was 0.73 the odds of an urban patient being a telehealth user (95% CI, 0.71-0.75; *P* < 0.01).

**Table 2:** Characteristics Associated With Being a Telehealth User

Covariate	OR (95% CI)	*P*
Rural (Ref: Urban)	4.61 (4.11-5.28)	<0.01
During COVID-19 (Ref: Pre-COVID–19)	139.55 (128.33-153.94)	<0.01
Rural × COVID-19	0.16 (0.14-0.18)	<0.01
Age (Ref: ≥80)		
0-19	1.38 (1.25-1.51)	<0.01
20-29	1.49 (1.41-1.58)	<0.01
30-39	1.46 (1.39-1.54)	<0.01
40-49	1.36 (1.29-143)	<0.01
50-59	1.27 (1.21-1.33)	<0.01
60-69	1.17 (1.12-1.22)	<0.01
70-79	1.09 (1.04-1.14)	<0.01
Female (Ref: Male)	1.19 (1.16-1.21)	<0.01
Race (Ref: White)		
African American	0.73 (0.72-0.75)	<0.01
Native American/American Indian	1.04 (0.96-1.12)	0.29
Native Hawaiian, Pacific Islander	0.92 (0.84-1.00)	0.06
Other	1.04 (1.00-1.08)	0.06
Hispanic (Ref: Not Hispanic)	1.04 (1.01-1.08)	<0.01
Primary disease (Ref: Diabetes)		
Cystic disease	0.96 (0.92-1.01)	0.09
Glomerulonephritis	1.01 (0.98-1.04)	0.42
Hypertension	0.86 (0.84-0.89)	<0.01
Urologic	0.87 (0.82-0.94)	<0.01
Other cause	1.08 (1.04-1.11)	<0.01
Unknown cause	0.93 (0.87-0.98)	0.01
Missing cause	0.99 (0.42-1.79)	0.97
Last kidney replacement therapy modality (Ref: Hemodialysis)		
Home hemodialysis	1.59 (1.52-1.67)	<0.01
In-center self-hemodialysis	1.59 (1.26-1.97)	<0.01
Peritoneal dialysis	1.37 (1.34-1.41)	<0.01
Employment status (Ref: Full-time)		
Medical leave of absence	1.00 (0.96-1.05)	0.86
Other	0.96 (0.90-1.01)	0.10
Part-time	0.99 (0.93-1.04)	0.60
Retired	1.02 (-0.99-1.05)	0.26
Student	1.12 (1.02-1.25)	0.02
Unemployed	0.94 (0.91-0.97)	<0.01
Institutionalized (Ref: Yes)	0.91 (0.87-0.96)	<0.01
County social vulnerability index (Ref: Q1)		
Quartile 2 (≥0.25 to <0.50)	1.10 (1.06-1.14)	<0.01
Quartile 3 (≥0.50 to ≤0.75)	1.05 (1.01-1.09)	<0.01
Quartile 4 (>0.75) [low]	1.12 (1.08-1.15)	<0.01

*Note:* Results are reported as estimated adjusted ORs (and 95% CIs) from a multivariable logistic regression model with the outcome as being a telehealth user (1 for user, 0 for nonuser) and the following predictors: urban vs rural patient address, pre-COVID–19 (January 2020) vs during COVID-19 (January 2021), an interaction between these 2 predictors to examine whether urban–rural differences changed during COVID-19 compared with pre-COVID–19, as well other patient characteristics including age, sex, race, ethnicity, primary disease, first kidney replacement therapy modality, employment status, institutionalization status, and CDC/ATSDR social vulnerability index. Because some patients appear in both cohorts, bootstrapped errors were used for hypothesis testing. Abbreviations: CDC/ATSDR, Centers for Disease Control and Prevention/Agency for Toxic Substances and Disease Registry; CI, confidence interval; COVID-19, coronavirus disease 2019; OR, odds ratio; Ref, reference.

There were some notable disparities in characteristics of telehealth users. Being a telehealth user was associated with younger age, and women were more likely to use telehealth than men (OR, 1.19; 95% CI, 1.16-1.21; *P* < 0.01), whereas African American (OR, 0.73; 95% CI, 0.72-0.75; *P* < 0.01) were less likely to use telehealth than White patients. Patients of Hispanic ethnicity were more likely to be users (OR, 1.04; 95% CI, 1.01-1.08; *P* < 0.01). Compared with in-center hemodialysis, use of in-center self-hemodialysis (OR, 1.59; 95% CI, 1.26-1.97; *P* < 0.01), home hemodialysis (OR, 1.59; 95% CI, 1.52-1.67; *P* < 0.01), and peritoneal dialysis (OR, 1.37; 95% CI, 1.34-1.41; *P* < 0.01) were associated with being a telehealth user. Telehealth utilization was modestly more likely among patients living in less socially vulnerable counties, with higher odds observed for those in quartiles 3 and 4 than the most vulnerable quartile.

Results from the sensitivity analysis, when accounting for COVID-19 prevalence, demonstrated directionally similar results. Being a patient in a rural county was associated with a higher likelihood of being telehealth user (OR, 4.76; 95% CI, 4.25-5.45; *P* < 0.01) compared with patients residing in an urban county. In January 2021, the odds of a rural patient being a telehealth user was 0.73 (95% CI, 0.71-0.75) the odds of an urban patient being a telehealth user. Finally, being a patient in a COVID-19 prevalent county significantly increased the likelihood of being a telehealth user (OR, 1.26; 95% CI, 1.22-1.30; *P* < 0.01, Table S3), but had minimal effect on the main predictors of interest.

### Geographic Variation in Telemedicine Use

**Figure 3:**
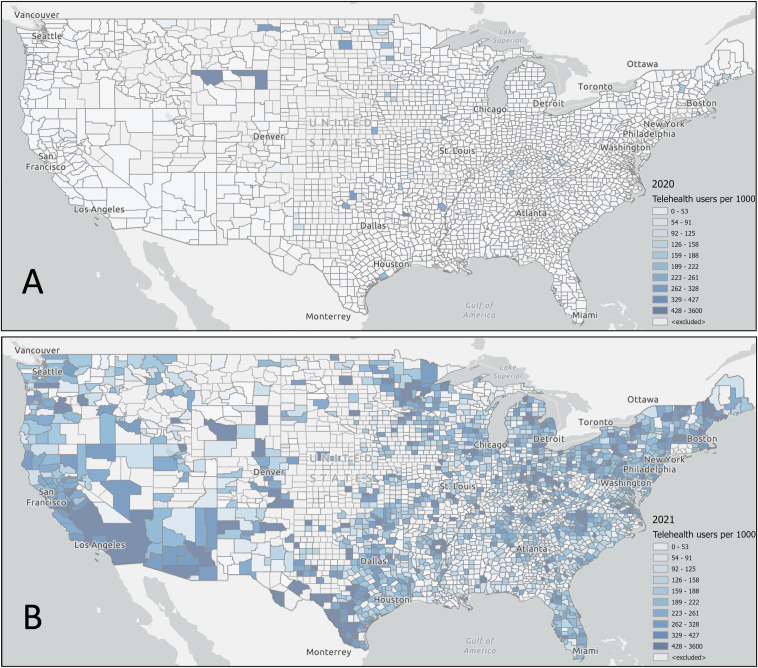

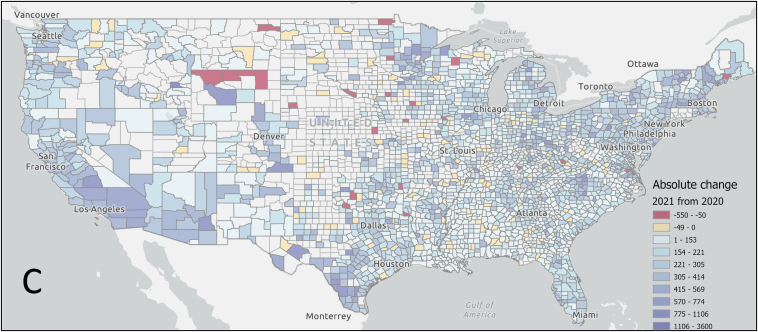
Telehealth utilization by county in January 2020 and January 2021. Pre-COVID–19 (January 2020, panel A) and during COVID-19 (January 2021, panel B) county prevalence of telehealth utilization per 1,000 patients with kidney failure. Because some counties had decreases in utilization, the absolute differences are shown in panel C. Patients were considered telehealth users if they had ≥1 claim with a telehealth modifier in either January 2020 or 2021. Counties are excluded (blank) if they had <11 patients with kidney failure. COVID-19, coronavirus disease 2019.

Uptake in telehealth across the country showed significant growth between January 2020 (Fig 3A) and January 2021 (Fig 3B). At the county level, the median number of telehealth users per 1,000 patients with kidney failure was 0 (0-0, 25th to 75th percentile) in January 2020. The median number of telehealth users increased to 108.1 (185.0-285.7) in January 2021. Areas of the country that showed the most absolute growth in telehealth utilization (Fig 3C) were in concentrated in larger, urban areas; several rural counties in the middle of the country demonstrated an absolute decrease in telehealth users per 1,000 patients with kidney failure.

## Discussion

The COVID-19 pandemic accelerated telemedicine adoption in care for patients with kidney failure through regulatory shifts, including expanded Medicare reimbursement.^12^ Utilization increased significantly in both rural and urban areas, although urban populations saw greater relative growth, possibly because of higher provider availability and better digital infrastructure (Fig 1). Rural patients, who already relied on telemedicine to overcome geographic barriers, continued to use it at higher rates than prepandemic. As pandemic-era flexibilities expire, ensuring sustained access to telemedicine, particularly for rural populations, remains a critical policy priority.

Telehealth use varied by both service type and geographic setting (Fig 2). In urban areas, the highest telehealth use was for “other” services, followed by nephrology, primary care, and mental health. In contrast, rural patients most commonly used telehealth for nephrology, followed by “other” services, primary care, and mental health. These differences suggest that both service availability and geographic context influence utilization patterns, rather than service availability alone. Notably, telemedicine use for mental health services remained relatively stable over time for both rural and urban patients, in contrast to the sharp post-2020 declines observed in other service categories. This durability underscores telehealth’s critical role in sustaining behavioral health access and highlights telemedicine’s versatility in meeting population needs—overcoming distance in rural areas and reducing exposure risk in urban centers.

Despite its benefits, telemedicine access remains inequitable. African American patients consistently demonstrated lower rates of telemedicine use than White patients. These disparities are likely influenced by multiple factors, including broadband limitations, lower digital literacy, provider biases in telehealth referrals, and language barriers. Area-level social disadvantage, as measured by the CDC/ATSDR social vulnerability index, was also associated with lower telehealth use, suggesting that community-level vulnerabilities—including economic instability, housing, or transportation barriers—may influence the capacity to engage with telemedicine, even within insured populations. Addressing these gaps requires expanding broadband access in underserved areas, culturally tailored patient education, and provider training to reduce implicit biases that may limit telehealth referrals.

Broadband access remains a significant barrier for rural patients, despite their reliance on telemedicine. However, patients with kidney failure receiving in-center hemodialysis may face fewer digital access barriers, because many dialysis facilities provide internet-enabled devices and infrastructure to support virtual visits during treatment sessions. This clinical environment reduces the impact of certain social determinants of health by facilitating provider-patient communication without requiring travel or patient-owned technology. An estimated 22% of rural households lack high-speed internet,^23^ making video-based telehealth difficult or impossible for many patients. Even with internet access, older adults and those with limited digital literacy often struggle with telehealth platforms.

Expanding broadband infrastructure, investing in digital literacy programs, and improving telehealth usability will be critical to sustaining equitable access. Federal programs such as the Rural Digital Opportunity Fund, the CARES Act, and the American Rescue Plan Act have provided substantial funding to expand broadband infrastructure and telehealth capabilities, particularly in rural and underserved communities. These initiatives, alongside efforts to improve digital literacy and subsidize patient technology access, are essential to bridging the digital divide and enabling broader engagement with telemedicine services.

Several policy measures are necessary to sustain telemedicine access for patients with kidney failure. Permanent Medicare reimbursement for telehealth services is essential to prevent disruptions, and payment parity between telehealth and in-person visits will support continued provider participation. Broadband expansion is critical to ensure rural patients have the infrastructure needed for virtual care. Digital literacy programs and telehealth navigation support should be prioritized to improve patient engagement, particularly among older adults and minority populations. Expanding interpreter services, developing culturally tailored outreach initiatives, and increasing provider awareness of disparities in telehealth referrals are also necessary to ensure equitable adoption. Investments in community-based telemedicine initiatives and subsidized technology access could further promote widespread utilization.

Further research is needed to assess whether telemedicine improves health care access and outcomes in care for patients with kidney failure. Studies examining its effects on hospitalization rates, transplant evaluations, and long-term disease management will help determine its clinical impact. The role of permanent telehealth reimbursement policies in shaping provider behavior and patient engagement should also be explored. Evaluating hybrid care models—in which telehealth complements in-person visits—will be critical in shaping future health care delivery models.

A key limitation of this study is its focus on traditional Medicare beneficiaries, excluding patients enrolled in Medicare Advantage (MA) and private insurance plans. Given that a growing proportion of patients with kidney failure are covered under MA, future studies should incorporate MA claims to provide a more comprehensive analysis of telemedicine utilization and access. Differences in telehealth coverage and provider networks across MA plans may influence adoption patterns. Additionally, this study did not evaluate patient outcomes related to telehealth use, such as hospitalization rates or transplant evaluations, which are critical to understand the long-term impact of telemedicine in care for patients with kidney failure. Although we updated kidney failure treatment modality to reflect current treatment, some patient characteristics—such as comorbid conditions and employment status—were captured only at dialysis initiation and may not reflect patients’ status at the time of telemedicine use.

Telemedicine has transformed care for patients with kidney failure, expanding access for rural and underserved populations while providing an alternative to in-person visits. However, disparities in adoption, broadband limitations, and digital literacy gaps persist. Addressing these barriers through policy reforms and infrastructure investments will ensure that telemedicine remains a viable, equitable component of kidney disease care. Future efforts should focus on integrating telemedicine into comprehensive care models, making its benefits accessible to all patient populations.
