# Supplementary material for: Differences in Telemedicine Use Between Rural and Urban Medicare Beneficiaries With Kidney Failure
Source: Kidney Med. 2026 Mar 20;8(5):101329. doi: 10.1016/j.xkme.2026.101329 (PMC13092677; doi:10.1016/j.xkme.2026.101329)
Supplement: Supplementary (PDF) — Table S1-S3. [file mmc1.pdf]

Table S1: Diagnosis codes used to assign nurse practitioner and physician assistant Medicare claims in USRDS

Diagnosis Codes	Provider Specialty Codes	Classification
F10x~F69x, F80x~F99x		Mental health
N186, Z940		Nephrology
None above	01, 08, 11, 37, 38, 42, 84	Primary Care
None above	39	Nephrology
N179, N184, N185, N1830, Z8422, Z944, I120, I129	50, 97	Nephrology
All else		Other

Table S2: Characteristics of utilizers and non-utilizers of telehealth, before and during Covid19

Covariate	Jan-20				Jan-21			
	User		Non-User		User		Non-User	
	n =1,035	%	n=369,866	%	n=57,205	%	n=259,192	%
County RUCA code								
Urban	503	0.17%	302,917	99.8%	48,628	18.8%	210,389	81.2%
Rural	532	0.79%	66,949	99.2%	8,577	14.9%	48,803	85.1%
Age (years)								
0-19	25	0.40%	6,269	99.6%	1,227	21.5%	4,480	78.5%
20-29	42	0.25%	16,955	99.8%	3,217	21.0%	12,087	79.0%
30-39	96	0.28%	33,599	99.7%	5,856	20.1%	23,314	79.9%
40-49	169	0.29%	57,596	99.7%	9,028	18.9%	38,839	81.1%
50-59	234	0.27%	84,993	99.7%	12,618	18.1%	56,911	81.9%
60-69	245	0.27%	90,821	99.7%	13,672	17.6%	64,152	82.4%
70-79	158	0.28%	57,014	99.7%	8,498	16.7%	42,383	83.3%
>= 80	66	0.29%	22,609	99.7%	3,089	15.4%	17,026	84.6%
Sex								
Female	490	0.31%	158,611	99.7%	26,264	19.4%	108,809	80.6%
Male	545	0.26%	211,255	99.7%	30,941	17.1%	150,383	82.9%
Race (n, %)								
Black	245	0.20%	123,079	99.8%	37,256	30.7%	84,138	69.3%
Native American/American Indian	60	1.15%	5,163	98.9%	831	18.1%	3,758	81.9%
Native Hawaiian, Pacific Islander	8	0.19%	4,194	99.8%	692	18.9%	2,973	81.1%
Other	27	0.16%	17,299	99.8%	3,311	20.7%	12,708	79.3%
White	695	0.31%	220,131	99.7%	37,256	19.3%	155,615	80.7%
Ethnicity (n, %)								
Hispanic or Latino	95	0.17%	54,303	99.8%	9,494	20.6%	36,590	79.4%
Not Hispanic or Latino	940	0.30%	315,563	99.7%	47,711	17.7%	222,602	82.3%

Cause of kidney failure								
Cystic disease	35	0.26%	13,547	99.7%	2,435	19.8%	9,836	80.2%
Diabetes mellitus	501	0.31%	159,385	99.7%	24,494	18.4%	108,447	81.6%
Glomerulonephritis	109	0.25%	44,037	99.8%	7,980	20.6%	30,841	79.4%
Hypertension	233	0.21%	108,404	99.8%	14,550	15.8%	77,515	84.2%
Urologic	22	0.33%	6,705	99.7%	1,032	17.3%	4,935	82.7%
Other	111	0.37%	29,497	99.6%	5,264	20.0%	20,994	80.0%
Unknown	23	0.28%	8,242	99.7%	1,440	18.0%	6,574	82.0%
Missing cause	**	**	**	**	**	**	**	**
First dialysis modality (n, %)								
Home hemodialysis	18	0.17%	10,312	99.8%	2,401	25.8%	6,897	74.2%
In-center self-hemodialysis	**	**	346	**	100	0.04%	223,175	99.96%
In-center hemodialysis	939	0.29%	321,337	99.7%	46,074	99.4%	295	0.6%
Peritoneal dialysis	77	0.20%	37,871	99.8%	8,630	23.0%	28,825	77.0%
Employment status (n, %)								
Full time	101	0.21%	48,804	99.8%	8,470	19.3%	35,472	80.7%
Medical leave of absence	35	0.22%	16,214	99.8%	2,668	19.1%	11,288	80.9%
Other	37	0.27%	13,616	99.7%	2,231	20.0%	8,920	80.0%
Part-time	35	0.28%	12,468	99.7%	2,070	19.0%	8,852	81.0%
Retired	549	0.32%	173,247	99.7%	25,994	17.5%	122,539	82.5%
Student	25	0.60%	4,172	99.4%	870	22.8%	2,952	77.2%
Unemployed	253	0.25%	101,345	99.8%	14,902	17.7%	69,169	82.3%
Institutionalized (n, %)	90	0.61%	14,707	99.4%	2,094	17.6%	9,825	82.4%
County social vulnerability index (n, %)								
Quartile 1 (<0.25) [high]	169	0.49%	34,462	99.5%	5,389	17.2%	26,008	82.8%
Quartile 2 (>= 0.25, < 0.50)	258	0.35%	74,307	99.7%	12,177	18.6%	53,258	81.4%
Quartile 3 (>= 0.50, <= 0.75)	212	0.21%	100,365	99.8%	15,352	17.9%	70,460	82.1%
Quartile 4 (>= 0.75) [low]	396	0.25%	160,732	99.8%	24,287	18.2%	109,466	81.8%

Table displays row/month percentages (i.e. the distribution within each covariate category and the month). For example, among individuals in the Jan 2020 cohort who had an urban RUCA code, 0.17% of them used telehealth in this month and 99.8% of them did not use telehealth in this month. Among individuals in the Jan 2021 cohort who had an urban RUCA code, 18.8% of them used

telehealth in this month and 81.2% of them did not use telehealth this month. ** values suppressed if the number of individuals is less than 11 in a cell, or if it could be calculated from those values

Table S3: Sensitivity analysis when accounting for Covid19 cases within county

Covariate	Odds Ratio (95% C.I.)	P value
Rural (Ref: Urban)	4.76 (4.25-5.45)	<0.01
During Covid (Ref: Pre-Covid)	141.53 (129.81-156.03)	<0.01
Rural*Covid	0.15 (0.13-0.17)	<0.01
Age (Ref: >=80)		
0-19	1.38 (1.25-1.51)	<0.01
20-29	1.50 (1.41-1.59)	<0.01
30-39	1.47 (1.39-1.54)	<0.01
40-49	1.37 (1.30-1.43)	<0.01
50-59	1.27 (1.21-1.34)	<0.01
60-69	1.18 (1.13-1.23)	<0.01
70-79	1.09 (1.04-1.15)	<0.01
Female (Ref: Male)	1.19 (1.16-1.21)	<0.01
Race (Ref: White)		
Black	0.74 (0.72-0.76)	<0.01
Native American/American Indian	1.00 (0.93-1.09)	0.96
Native Hawaiian, Pacific Islander	1.03 (0.99-1.07)	0.17
Other	0.93 (0.85-1.00)	0.08
Hispanic (Ref: Not Hispanic)	1.02 (1.00-1.05)	0.10
Primary disease (Ref: Diabetes)		
Cystic disease	0.96 (0.92-1.01)	0.12
Glomerulonephritis	1.02 (0.99-1.05)	0.30
Hypertension	0.86 (0.85-0.89)	<0.01
Urologic	0.88 (0.82-0.95)	<0.01
Other cause	1.07 (1.04-1.11)	<0.01
Unknown cause	0.92 (0.86-0.98)	<0.01
Missing cause	0.99 (0.43-1.79)	0.97
First dialysis modality (Ref: Hemodialysis)		
Home hemodialysis	1.60 (1.53-1.68)	<0.01
In-center self-hemodialysis	1.58 (1.24-1.98)	<0.01
Peritoneal dialysis	1.38 (1.34-1.42)	<0.01
Employment status (Ref: Full-time)		
Medical leave of absence	1.00 (0.95-1.05)	0.99
Other	0.95 (0.90-1.00)	0.07
Part-time	0.99 (0.93-1.04)	0.68
Retired	1.02 (0.99-1.05)	0.27
Student	1.13 (1.02-1.24)	0.02
Unemployed	0.93 (0.91-0.96)	<0.01
Institutionalized (Ref: Yes)	0.92 (0.87-0.97)	<0.01
County social vulnerability index (Ref: Q1)		
Quartile 2 (>= 0.25, < 0.50)	1.09 (1.05-1.13)	<0.01

Quartile 3 (>= 0.50, <= 0.75)	1.03 (0.99-1.06)	0.11
Quartile 4 (> 0.75) [low]	1.08 (1.04-1.12)	<0.01
Counties with Covid19 prevalence (Ref: Low)	1.26 (1.22-1.30)	<0.01
